# Prognostic Value of the Overexpression of Fatty Acid Metabolism-Related Enzymes in Squamous Cell Carcinoma of the Head and Neck

**DOI:** 10.3390/ijms21186851

**Published:** 2020-09-18

**Authors:** Ying-Wen Su, Pao-Shu Wu, Sheng-Hsiang Lin, Wen-Yu Huang, Yu-Shao Kuo, Hung-Pin Lin

**Affiliations:** 1 Division of Hematology and Medical Oncology, Department of Internal Medicine, MacKay Memorial Hospital, Taipei 10449, Taiwan; yingwensu.5896@mmh.org.tw; 2 Department of Pathology, MacKay Memorial Hospital, Tamsui Branch, New Taipei City 25160, Taiwan; 3 MacKay Junior College of Medicine, Nursing, and Management, Taipei 25245, Taiwan; 4 Institute of Clinical Medicine and Department of Public Health, College of Medicine, National Cheng Kung University, Tainan 70101, Taiwan; shlin922@mail.ncku.edu.tw; 5 Biostatistics Consulting Center, National Cheng Kung University Hospital, College of Medicine, National Cheng Kung University, Tainan 70101, Taiwan; 6 Laboratory of Good Clinical Research Center, MacKay Memorial Hospital, Tamsui Branch, New Taipei City 25160, Taiwan; b96608036@ntu.edu.tw; 7 School of Chinese Medicine, China Medical University, Taichung 40402, Taiwan; u102023701@cmu.edu.tw; 8 Division of Oral Pathology, Department of Stomatology, MacKay Memorial Hospital, Taipei 10449, Taiwan

**Keywords:** fatty acid metabolism, head and neck, squamous cell carcinoma, long-chain acyl-CoA dehydrogenase

## Abstract

Reprogramming of cellular energy metabolism, such as lipid metabolism, is a hallmark of squamous cell carcinoma of the head and neck (SCCHN). However, whether protein expression related to fatty acid oxidation (FAO) affects survival in SCCHN remains unclear. We aimed to investigate FAO-related enzyme expression and determine its correlation with clinicopathological variables in SCCHN patients. Immunohistochemical analysis (IHC) of FAO-related protein expression, including carnitine palmitoyltransferase 1 (CPT1), the acyl-CoA dehydrogenase family, and fatty acid synthase (FAS), was performed using tissue microarrays from 102 resected SCCHN tumors. Expressions were categorized according to IHC scores, and the statistical association with clinicopathological factors was determined. Moderate-to-high expression of long-chain acyl-CoA dehydrogenase (LCAD) had a protective role against cancer-related death (adjusted hazard ratio (HR), 0.2; 95% confidence interval (CI), 0.05–0.87) after covariate adjustment. Age and clinical stage remained independent predictors of survival (adjusted HR, 1.75; 95% CI, 1.22–2.49 for age; adjusted HR, 14.33; 95% CI, 1.89–108.60 for stage III/IV disease). Overexpression of medium-chain acyl-CoA dehydrogenase and FAS correlated with advanced tumor stage (T3/T4); however, none of these factors were independent predictors of survival. Several FAO-related enzymes were upregulated and LCAD overexpression had a protective effect on overall survival in advanced SCCHN patients. FAO-related-enzyme expression might have a prognostic impact on survival outcomes in SCCHN.

## 1. Introduction

Squamous cell carcinoma of the head and neck (SCCHN) is one of the leading causes of cancer-related death globally [1]. One of the hallmarks of SCCHN is the reprogramming of cellular energy metabolism [2,3]. SCCHN tumors expressing a high level of phosphorylated acetyl-CoA carboxylase 1 (pACC1) or ACC2 are associated with decreased survival [4,5]. The major cellular function of ACC is to produce malonyl-CoA from acetyl-CoA for fatty acid synthesis [6]. Phosphorylation of ACC inhibits catabolic functions such as lipid storage and causes cellular metabolism to switch to fatty acid oxidation (FAO) [7]. FAO is a catabolic process by which fatty acid molecules are broken down enzymatically. In mammalian cells, FAO primarily occurs in mitochondria except for the oxidation of very-long-chain fatty acids, which occurs in peroxisomes [8]. The major enzymes involved in FAO include carnitine palmitoyltransferase (CPT), the acyl-CoA dehydrogenase family, and hydroxyacyl-CoA dehydrogenase/3-ketoacyl-CoA thiolase/enoyl-CoA hydratase (HADHA). The acyl-CoA dehydrogenase (ACAD) family includes very-long-chain, long-chain, medium-chain, and short-chain acyl-CoA dehydrogenases (VLCAD, LCAD, MCAD, and SCAD, respectively) [9]. In addition to useful intermediates, the final product of FAO is acetyl-CoA, which enters the citric acid cycle in the mitochondrion for energy generation.

Despite the negative correlation of pACC1 or ACC2 expression with clinical outcome [4,5], it remains unclear whether the expression of the enzymes involved in FAO is associated with cancer progression in SCCHN. Therefore, this study aimed to retrospectively examine the expressions of major enzymes involved in FAO in patients with resected SCCHN and determine the correlation with clinicopathological variables.

## 2. Results

### 2.1. Incidence of Expression of Fao-Related Proteins in Scchn Tumors and Patient Characteristics According to Survival Status, Age, and Clinical Stage

A total of 102 patients with SCCHN were included in the study between 1 January 2011, and 31 December 2012. The median follow-up time was 5.1 years (interquartile range (IQR), 2.6–6.1). Twenty-nine patients died during the study period. The clinicopathological characteristics of the patients are listed in Table 1.

Compared with patients who died during the study period, patients who survived were significantly younger (50.64 vs. 56.09 years, *p* = 0.026, Mann–Whitney U test), did not have lymph node involvement (54.79% vs. 31.03%, *p* = 0.032, χ^2^ test), and were more likely to experience stage I/II disease (30.14% vs. 3.45%, *p* = 0.004, χ^2^ test) (Table 2).

We next examined the expressions of FAO-related proteins according to age. None (0%) of the 28 patients aged ≥60 years showed a high expression of CPT1A (3+ score), whereas 14 (18.92%) out of 74 patients aged <60 years exhibited a high expression of CPT1A (3+ score) (*p* = 0.01, Fischer’s exact test). However, the negative correlation between CPT1A and age was not further demonstrated by the Spearman correlation analysis (Spearman coefficient for CPT1A and age: −0.09, *p* = 0.35). There were no statistically significant differences in the levels of pretreatment fasting sugar, cholesterol and triglycerides between both age groups (Table S1).

In terms of clinical stage (Table 3), the incidence of a high expression of MCAD (3+ score) was significantly higher among patients with a more-advanced SCCHN (stage III/IV) (33 of 79 patients, 41.77%) than among patients with stage I/II disease (2 of 23 patients, 8.70%) (*p* = 0.01, Fisher’s exact test). The incidence of high LCAD expression (2+ to 3+ score) was also higher in patients with stage III/IV disease (15 of 79 patients, 18.99%) than in patients with state I/II disease (1 of 23 patients, 4.35%), but the differences were not statistically significant (*p* = 0.11, χ^2^ test). Moreover, patients with stage III/IV disease had significantly higher levels of pretreatment fasting sugar than did patients with stage I/II disease (median: 99.00 vs. 92.00 mg/dl, *p* = 0.01, Mann–Whitney U test). However, no significant differences in cholesterol and triglyceride levels were observed between patients with stage I/II disease and patients with stage III/IV disease.

Regarding clinical T status (Table 4), patients with a higher T stage (T3/T4) were more likely to have a moderate to high FAS expression (2+ to 3+ score) and high MCAD expression (3+ score) than patients with a lower T stage (T1/T2) (FAS: 12 of 58 patients with T3/T4 stage (20.69%) vs. 2 of 44 patients with T1/T2 stage (4.55%), *p* = 0.04, Fisher’s exact test; MCAD: 27 of 58 patients with T3/T4 stage (46.55%) vs. 8 of 44 patients with T1/T2 stage (18.18%), *p* = 0.01, χ^2^ test). Meanwhile, the incidence of increased expressions of CPT1 (3+ score) was high among patients with T3/T4 stage (12 of 58 patients, 20.69%) than among patients with T1/T2 stage (3 of 44 patients, 6.82%), but the difference was not statistically significant (*p* = 0.14, Fisher’s exact test). There were no statistically significant differences in the above parameters between the node-negative and node-positive subgroups (Table S2).

We also examined the correlation of FAO-related enzyme expression with either human papillomavirus (HPV) infection status or tumor proliferation activity. As shown in Tables S3 and S4, there was no statistically significant difference of FAO-related protein expression in HPV-positive vs. HPV-negative tumor or in tumor with high vs. low proliferation index (Ki-67 ≥ 10% vs. Ki-67 < 10%).

### 2.2. Univariate and Multivariable Cox Regression Analyses

In the univariate Cox regression analysis, increased age and advanced stage were significantly correlated with poor survival outcomes (crude hazard ratio (HR), 1.49 (95% confidence interval (CI), 1.11–1.98) and 9.59 (95% CI, 1.30–70.51), respectively) (Table 5). We ran a multicollinearity analysis, which showed no collinearity among the covariates (variance inflation factors (VIF) < 1.8, Table 5). After multivariate adjustment, the risk for cancer-related death remained high with increased age and advanced clinical stage (adjusted HR, 1.75 (95% CI, 1.22–2.49) and 14.33 (95% CI, 1.89–108.60), respectively). Moreover, overexpression of LCAD (2+ to 3+ score) had an independent protective role against cancer-related death (adjusted HR, 0.2; 95% CI, 0.05–0.87).

In patients with stage III/IV SCCHN (*n* = 78), the risk of death was high among those with advanced age (adjusted HR, 1.59; 95% CI, 1.10–2.29) but low among those who had a moderate to high expression of LCAD (adjusted HR, 0.21; 95% CI, 0.05–091) (Table 6). However, the HRs of death did not differ significantly in terms of overexpression of other FAO-related proteins such as MCAD in patients with stage III/IV disease.

Similar results were observed in patients with T3/T4 stage (*n* = 58). The risk of death was high among those with advanced age (adjusted HR, 1.92; 95% CI, 1.16–3.18) but low among those who had a moderate to high expression of LCAD (adjusted HR, 0.09; 95% CI, 0.01–085) (Table 7).

## 3. Discussion

In this retrospective study, we found that moderate to high expression of LCAD had a protective role against cancer-related death after adjustment for clinical variables such as age and stage. Overexpression of MCAD and FAS were correlated with an advanced tumor stage (T3/T4), however, none of these factors were independent predictors of survival.

Previous studies on cellular metabolism in SCCHN have revealed dysregulation in multiple metabolic pathways in tumors, such as the mitochondrial oxidative phosphorylation and the tricarboxylic acid cycle [10], and in the utilization of glucose as a dominant energy source for the survival and proliferation of SCCHN cells [11]. Despite the endeavors in targeting aerobic glycolysis in the treatment of SCCHN, no glycolytic inhibitors have been used in the clinical setting so far [12], suggesting that SCCHN cells might have alternative metabolic pathways to acquire energy to survive. Our previous study, and a study by another group, have shown that overexpression of phosphorylated inhibitory ACC1 or ACC2 was associated with a decreased survival rate in SCCHN [4,5]. Since the major function of ACC1 is to regulate fatty acid metabolism, and ACC2 regulates FAO [13,14], the reprograming of lipid metabolism may play a role in tumor progression.

LCAD is a mitochondrial enzyme that catalyzes the initial step of FAO [15]. Although LCAD belongs to the ACAD protein family, the exact physiological role of LCAD in FAO remains to be elucidated. In humans, VLCAD, MCAD, and SCAD are specific to large, medium, and short chain acyl-CoAs, respectively. In contrast, LCAD has broad substrate specificity and is active with not only medium and long chain acyl-CoAs but also branched-chain acetyl CoA [16]. The overlapped substrate activity suggests that LCAD might play less-important roles in FAO in humans [17,18]. Recently, Zhang et al. showed a unique role of LCAD in FAO-driven oxidative signaling, which does not exist in other ACAD family members [19]. This suggests LCAD may have a distinct function in control of cell proliferation. Despite using different scoring systems, immunohistochemistry (IHC) studies have found a high expression of LCAD in 45–50% of patients with hepatocellular carcinoma (HCC) [20,21] and in 48.9% of patients with esophageal squamous cell carcinoma [22]. A low expression or methylation of LCAD has been associated with poor survival outcomes in several cancers such as HCC and breast cancer [20,21,23]. At the cellular level, LCAD can be inhibited by sirtuin 3 acetylation or by hypoxia-inducible factor-1α under hypoxic conditions [20,24]. In HCC cells, loss of LCAD has been shown to promote tumor progression as a consequence of the activation of Yap [21] or reduction of phosphatase and tensin homolog [20], while overexpression of LCAD has been shown to inhibit tumor growth [20], indicating that LCAD has tumor-suppressive functions. This was supported by our data that LCAD had a protective role against cancer-related death (adjusted HR: 0.2; 95% CI, 0.05–0.87) after covariate adjustment (Table 5A). Furthermore, we also observed numerically increased expression of LCAD in patients with advanced stages (III and IV) of cancer (Table 3). However, this numeric difference was not statistically significant. Thus, selection bias or other confounding factors may have existed in the study cohort. This finding might explain the protective effect of LCAD expression in SCCHN, which was only observed after all covariate adjustment in the multivariate analysis. How LCAD is expressed in more patients with advanced disease but also exerts a protective role in their outcomes remains to be studied. Since only a small portion (19.23%) of advanced disease tissues overexpressed LCAD, whether such patients have different cellular control of the tumor proliferation rate, or sensitivity to chemotherapy or irradiation, needs to be investigated with larger clinical samples.

Our results also showed that the expression of MCAD and FAS increased as the tumor progressed from T1/T2 to T3/T4. MCAD also catalyzes mitochondrial FAO. MCAD deficiency is a potentially lethal inherited disease in neonates that may lead to hepatic dysfunction, fasting hypoglycemia, encephalopathy, or infant death [25]. Although the correlation between MCAD expression and cancer-related death was not observed in our multivariate analysis, a previous study has shown that a high expression of MCAD was associated with a better overall survival (OS) in neuroblastoma patients [26].

In this study, we also examined the expressions of FAS, an enzyme responsible for the endogenous synthesis of fatty acids, because several previous studies have shown that its overexpression was associated with poor prognosis, higher histologic grade, and nodal metastasis in SCCHN [27,28]. Consistent with the results of one of these studies [27], higher expressions of FAS in our patients was not an independent prognostic factor for survival after adjustment for other clinicopathological factors, despite its association with an advanced clinical T stage.

In summary, this study revealed that lipid metabolism is deranged in SCCHN, since several enzymes involved in FAO were upregulated in the advanced stage of the disease. In particular, we found that LCAD overexpression had a protective role for overall survival. The expression of these FAO-related enzymes might have a prognostic impact on survival outcomes in SCCHN. Although recent studies have shown that FAO is dysregulated in various tumor tissues [29] and that activating FAO will have a negative impact on tumor growth and progression, and will result in a more favorable clinical outcome [26,29,30,31], no in vitro study has determined the fate of SCCHN cells under an increased rate of FAO. Nevertheless, the present study was limited by its retrospective nature, small number of cases studied, and the semi-quantitative assessment of protein expression by immunohistochemistry. Further elucidation of the roles of FAO-related enzymes in SCCHN may hold promise for better management of the disease.

## 4. Materials and Methods

### 4.1. Ethics Statement

Samples were acquired after written informed consent was obtained from the participants. This study was approved by the Ethics Committee of Mackay Memorial Hospital (approval no. 16MMHIS070e and 18MMHIS197e).

### 4.2. Study Subjects and Tissue Microarray Construction

In this retrospective study, we collected data on 150 patients who underwent surgery for squamous cell carcinoma of the oral cavity, oropharynx, hypopharynx, or larynx at Mackay Memorial Hospital, Taiwan, between January 1, 2011, and December 31, 2012. We excluded patients whose tumors were recurrent (*n* = 23), who had other concurrent primary cancer (*n* = 7), who had prior exposure to neoadjuvant chemotherapy (*n* = 4), who did not receive definitive radical surgery (*n* = 9), who died within 2 months after surgery (*n* = 3), and who were lost to follow-up (*n* = 2). Therefore, 102 patients were included in the analysis. Formalin-fixed paraffin-embedded surgical tumor samples and paired normal mucosal tissues (3 μm thickness) were arrayed in quadruplicate for tissue microarray analysis. A chart review was conducted to retrieve clinicopathological information, including demographics, tumor-node-metastasis (TNM) stage, and OS. TNM staging was evaluated according to the guidelines of the American Joint Committee on Cancer, Eighth Edition (2017) [32]. Patients were monitored until death or until January 1, 2018, whichever was earlier. The baseline characteristics of the patients are listed in Table 1.

### 4.3. Immunohistochemistry and Scoring

**Figure 1:**
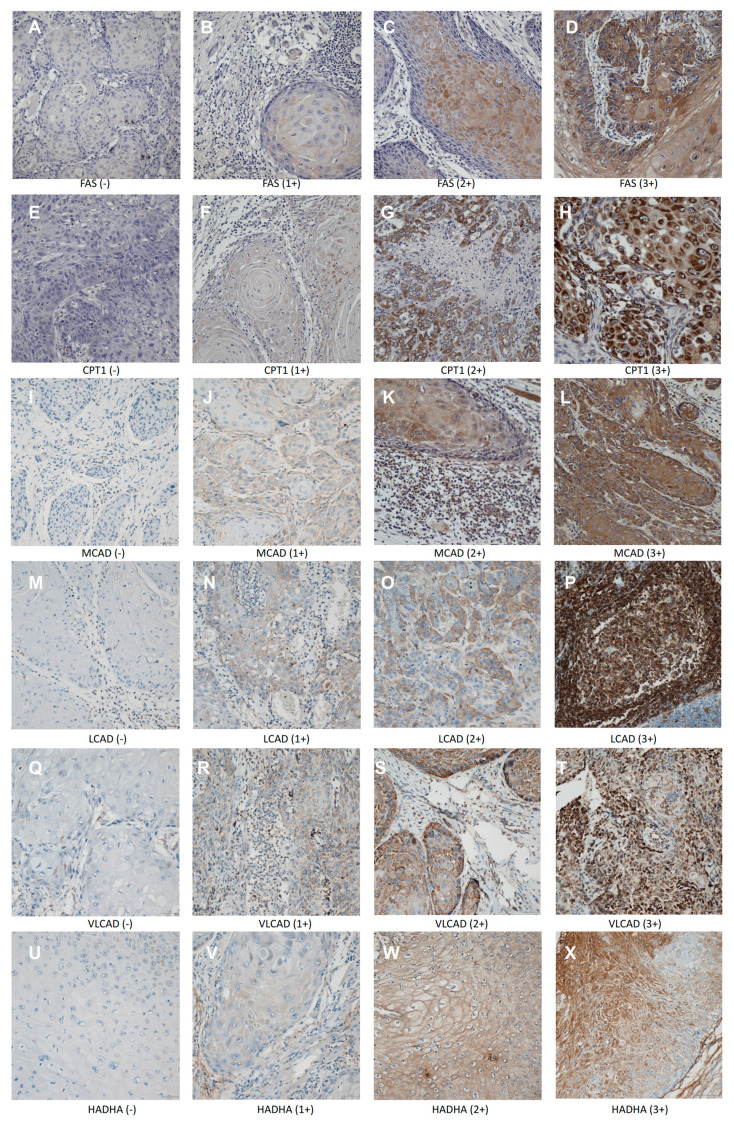
Representative immunohistochemical staining scores of antibodies used in the specimens of squamous cell carcinoma of the head and neck. All proteins were predominantly expressed in the cytoplasm of tumor cells. (**A**–**D**) fatty acid synthase (FAS) staining (0 to 3+), (**D**–**H**) carnitine palmitoyltransferase 1 (CPT1) staining (0 to 3+), (**I**–**L**) medium-chain acyl-CoA dehydrogenase (MCAD) staining (0 to 3+), (**M**–**P**) long-chain acyl-CoA dehydrogenase (LCAD) staining (0 to 3+), (**Q**–**T**) very-long-chain acyl-CoA dehydrogenase (VLCAD) staining (0 to 3+), and (**U**–**X**) hydroxyacyl-CoA dehydrogenase/3-ketoacyl-CoA thiolase/enoyl-CoA hydratase (HADHA) staining (0 to 3+), (magnification 200×).

Immunohistochemistry (IHC) of both tumor and paired normal mucosal tissues was performed as previously described [33]. Primary antibodies against FAS, CPT1A, MCAD, LCAD, VLCAD, and HADHA were obtained from Abcam (Cambridge, UK). Histopathological differentiation and expression of these proteins were evaluated independently by two pathologists (PS Wu and HP Lin) and quantified as previously described [4,33]. A composite score using the staining intensity (0, 1+, 2+, and 3+) and the percentage of the area showing reactivity was generated. Scores were averaged over replicate cores to obtain the final IHC score for each tumor. Representative examples of each score are presented in Figure 1. HPV p16 expression was evaluated with a CINtec p16 Histology kit following the manufacturer’s instructions (Ventana, Tucson, AZ, USA). Anti-Ki-67 antibody was obtained from Thermo Fisher Scientific (Waltham, MA, USA). Ki-67 expression was assessed based on percentage of positive nuclear staining in tumor regions.

### 4.4. Statistical Analysis

Baseline variables with a continuous outcome were expressed as medians and interquartile ranges (IQRs), and discrete variables as frequencies and percentages. To determine the associations between the expression of FAO-related enzymes and clinicopathological features, we performed the chi-squared (χ^2^) test or Fisher’s exact test to compare categorical variables and the Mann-Whitney U test to compare continuous variables. Baseline characteristics associated with cancer-related deaths were estimated using univariable Cox regression models and reported as crude hazard ratios (HRs) with 95% confidence intervals (CIs). To assess the presence of multicollinearity, variance-inflation factors (VIF) were checked. All VIFs for the covariates were low, indicating that no multicollinearity existed among these variables. To adjust for covariates, we calculated HRs by including independent baseline characteristics in the multivariable Cox regression model. All models incorporated cancer-related death as a dependent variable and baseline characteristics as independent variables, and the timeframe was from the date of the first anti-cancer treatment to the date of death or the last follow-up. All analyses were conducted using SAS software version 9.4 (SAS Institute, Cary, NC, USA). All statistical tests were two-sided, and significance was defined as a *p*-value of < 0.05.

## 5. Conclusions

In conclusion, we showed several enzymes involved in FAO were upregulated in patients with advanced SCCHN, whereas LCAD overexpression showed a protective role for overall survival in these patients. The expression of these FAO-related enzymes might have a prognostic impact on survival outcomes in SCCHN.

## Figures and Tables

**Table 1:** Characteristics of patients (*n* = 102).

Patient Characteristics	Number (%)
Age, median (range)	51.96 (30.3–86.5)
<60 years	74 (72.5%)
>60 years	28 (27.5%)
Gender	
Female	7 (6.9%)
Male	95 (93.1%)
Adjuvant treatment	
None	17 (16.7%)
Yes	85 (83.3%)
Site	
Non-oral cavity	13 (12.7%)
Oral cavity	89 (87.3%)
T	
1	13 (12.7%)
2	31 (30.4%)
3	23 (22.5%)
4	35 (34.3%)
N	
0	49 (48.0%)
1	19 (18.6%)
2	31 (30.4%)
3	3 (2.9%)
Stage	
I	8 (7.84%)
II	15 (14.71%)
III	25 (24.51%)
IV	54 (52.94%)
HPV P16	
Positive	13 (12.7%)
Negative	89 (87.3%)

T: tumor; N: node; HPV: Human papillomavirus.

**Table 2:** Correlations between the survival status and patients’ characteristics (*n* = 102).

Factor	Alive (*n* = 73)	Dead (*n* = 29)	*p*-Value ^a^
	*n* (%)	*n* (%)	
Age			
median (IQR)	50.64 (45.13, 57.02)	56.09 (48.61, 67.05)	0.026 *
Pretreatment fasting sugar			
<100 mg/dL	42 (57.53)	15 (51.72)	0.755
≥100 mg/dL	31 (42.47)	14 (48.28)	
median (IQR)	97.00 (91.00, 105.00)	96.00 (87.00, 118.00)	0.807
Pretreatment cholesterol			
<200 mg/dL	49 (67.12)	20 (68.97)	1.000
≥200 mg/dL	24 (32.88)	9 (31.03)	
median (IQR)	183.00 (156.00, 208.00)	172.00 (150.00, 202.00)	0.404
Pretreatment triglyceride			
<150 mg/dL	58 (79.45)	22 (75.86)	0.896
≥150 mg/dL	15 (20.55)	7 (24.14)	
median (IQR)	109.00 (84.00, 132.00)	98.00 (84.00, 129.00)	0.722
Stage			
I/II	22 (30.14)	1 (3.45)	0.004 *
III/IV	51 (69.86)	28 (96.55)	
T			
1–2	35 (47.95)	9 (31.03)	0.120
3–4	38 (52.05)	20 (68.97)	
N			
0	40 (54.79)	9 (31.03)	0.032 *
1–3	33 (45.21)	20 (68.97)	
FAS			
Negative or low expression (0~1+)	61 (83.56)	27 (93.10)	0.207
Moderate or strong expression (2~3+)	12 (16.44)	2 (6.90)	
CPT1			
Negative to moderate expression (0~2+)	63 (86.30)	25 (86.21)	0.990
Strong expression (3+)	10 (13.70)	4 (13.79)	
MCAD			
Negative to moderate expression (0~2+)	51 (69.86)	16 (55.17)	0.159
Strong expression (3+)	22 (30.14)	13 (44.83)	
LCAD			
Negative or low expression (0~1+)	59 (80.82)	27 (93.10)	0.124
Moderate or strong expression (2~3+)	14 (19.18)	2 (6.90)	
VLCAD			
Negative or low expression (0~1+)	65 (89.04)	23 (79.31)	0.198
Moderate or strong expression (2~3+)	8 (10.96)	6 (20.69)	
HADHA			
Negative to moderate expression (0~2+)	66 (90.41)	24 (82.76)	0.279
Strong expression (3+)	7 (9.59)	5 (17.24)	

^a^ χ^2^ test or Fisher’s exact test for categorical variables/Mann–Whitney U test for continuous variables. * *p* < 0.05; *n*: number; IQR: interquartile range; T: tumor; N: node; FAS: fatty acid synthase; CPT1: carnitine palmitoyl transferase 1; MCAD: medium-chain acyl-CoA dehydrogenase; LCAD: long-chain acyl-CoA dehydrogenase; VLCAD: very-long-chain acyl-CoA dehydrogenase; HADHA: hydroxyacyl-CoA dehydrogenase/3-ketoacyl-CoA thiolase/enoyl-CoA hydratase.

**Table 3:** Correlations between the clinical staging and patients’ characteristics (*n* = 102).

Factor	Stage I/II (*n* = 23)	Stage III/IV (*n* = 79)	*p*-Value ^a^
Age median (IQR)	52.64 (45.14, 63.30)	51.78 (45.13~61.03)	0.87
Pretreatment fasting sugar median (IQR)	92.00 (86.00, 102.00)	99.00 (88.00, 113.00)	0.01 *
Pretreatment cholesterol median (IQR)	180.00 (157.00, 201.00)	183.00 (148.00, 208.00)	0.95
Pretreatment triglyceride median (IQR)	109.00 (93.00, 152.00)	106.00 (83.00, 152.00)	0.50
FAS (2~3+) *n* (%)	2 (8.70%)	12 (15.19%)	0.73
CPT1(3+) *n* (%)	3 (13.0%)	11 (13.92%)	1.00
MCAD (3+) *n* (%)	2 (8.70%)	33 (41.77%)	0.01 *
LCAD (2~3+) *n* (%)	1 (4.35%)	15 (18.99%)	0.11
VLCAD (2~3+) *n* (%)	2 (8.70%)	12 (15.19%)	0.73
HADHA (3+) *n* (%)	2 (8.70%)	10 (12.66%)	1.00

^a^ χ ^2^ test or Fisher’s exact test for categorical variables/Mann-Whitney U test for continuous variables. * *p* < 0.05; *n*: number; IQR: interquartile range; T: tumor; N: node; FAS: fatty acid synthase; CPT1: carnitine palmitoyl transferase 1; MCAD: medium-chain acyl-CoA dehydrogenase; LCAD: long-chain acyl-CoA dehydrogenase; VLCAD: very-long-chain acyl-CoA dehydrogenase; HADHA: hydroxyacyl-CoA dehydrogenase/3-ketoacyl-CoA thiolase/enoyl-CoA hydratase.

**Table 4:** Correlations between T stage and patients’ characteristics (*n* = 102).

Factor	T1/T2 (*n* = 44)	T3/T4 (*n* = 58)	*p*-Value ^a^
Age median (IQR)	52.67 (47.87, 63.55)	51.59 (42.61, 58.43)	0.15
Pretreatment fasting sugar median (IQR)	94.00 (87.00, 104.50)	99.50 (91.00, 116.00)	0.08
Pretreatment cholesterol median (IQR)	188.50 (157.00, 206.00)	174.00 (147.00, 204.00)	0.30
Pretreatment triglyceride median (IQR)	110.00 (93.00, 151.50)	100.50 (81.00, 128.00)	0.13
FAS (2~3+) *n* (%)	2 (4.55%)	12 (20.69%)	0.04 *
CPT1(3+) *n* (%)	3 (6.82%)	11 (18.97%)	0.14
MCAD (3+) *n* (%)	8 (18.18%)	27 (46.55%)	0.01 *
LCAD (2~3+) *n* (%)	7 (15.91%)	9 (15.52%)	1.00
VLCAD (2~3+) *n* (%)	4 (9.09%)	10 (17.24%)	0.37
HADHA (3+) *n* (%)	3 (6.82%)	9 (15.52%)	0.30

^a^ χ^2^ test or Fisher’s exact test for categorical variables/Mann–Whitney U test for continuous variables. * *p* < 0.05; *n*: number; IQR: interquartile range; T: tumor; N: node; FAS: fatty acid synthase; CPT1: carnitine palmitoyl transferase 1; MCAD: medium-chain acyl-CoA dehydrogenase; LCAD: long-chain acyl-CoA dehydrogenase; VLCAD: very-long-chain acyl-CoA dehydrogenase; HADHA: hydroxyacyl-CoA dehydrogenase/3-ketoacyl-CoA thiolase/enoyl-CoA hydratase.

**Table 5:** Crude and adjusted HRs with 95% CIs for mortality related to clinical parameters and fatty acid oxidation-related expression of enzymes. Total case (*n* = 102).

Factor	Crude HR (95% CI)	*p*-Value	Adjusted HR (95% CI)	*p*-Value
Age, per 10 years	1.49 (1.11–1.98)	0.007 *	1.75 (1.22–2.49)	0.002 *
Pretreatment fasting sugar				
≥100 vs. <100 (mg/dL)	1.12 (0.54–2.31)	0.770	0.63 (0.29–1.37)	0.245
Stage: III/IV vs. I/II	9.59 (1.30–70.51)	0.026*	14.33 (1.89–108.60)	0.010 *
FAS: 2~3+ vs. 0~1+	0.41 (0.10–1.75)	0.232	0.33 (0.08–1.42)	0.135
MCAD: 3+ vs. 0~2+	1.75 (0.84–3.65)	0.133	1.30 (0.62–2.73)	0.495
LCAD: 2~3+ vs. 0~1+	0.36 (0.09–1.51)	0.161	0.20 (0.05–0.87)	0.032 *
VLCAD: 2~3+ vs. 0~1+	1.57 (0.64–3.85)	0.328	1.26 (0.49–3.21)	0.635
HADHA: 3+ vs. 0~2+	1.54 (0.58–4.04)	0.385	0.74 (0.27–2.01)	0.549

Variance inflation factors (VIF) values: Age: 1.32, Pretreatment fasting sugar: 1.08, Stage: 1.03, FAS: 1.34, MCAD: 1.55, LCAD: 1.28, VLCAD: 1.08, HADHA: 1.18. * *p* < 0.05.

**Table 6:** Crude and adjusted HRs with 95% CIs for mortality related to clinical parameters and fatty acid oxidation-related expression of enzymes. Stage III/IV case (*n* = 79).

Factor	Crude HR (95% CI)	*p*-Value	Adjusted HR (95% CI)	*p*-Value
Age, per 10 years	1.53 (1.10–2.12)	0.012 *	1.59 (1.10–2.29)	0.014 *
Pretreatment fasting sugar: ≥100 vs. <100 mg/dL	0.95 (0.45–1.99)	0.885	0.72 (0.33–1.56)	0.401
FAS: 2~3+ vs. 0~1+	0.17 (0.02–1.21)	0.077	0.15 (0.02–1.13)	0.066
MCAD: 3+ vs. 0~2+	1.34 (0.64–2.82)	0.439	1.36 (0.64–2.90)	0.431
LCAD: 2~3+ vs. 0~1+	0.28 (0.07–1.18)	0.084	0.21 (0.05–0.91)	0.037 *
VLCAD: 2~3+ vs. 0~1+	1.46 (0.59–3.60)	0.415	1.41 (0.54–3.64)	0.483
HADHA: 3+ vs. 0~2+	1.44 (0.55–3.80)	0.460	0.80 (0.29–2.20)	0.668

VIF values: Age: 1.33, Pretreatment fasting sugar: 1.08, FAS: 1.34, MCAD: 1.60, LCAD: 1.29, VLCAD: 1.09, HADHA: 1.18. * *p* < 0.05.

**Table 7:** Crude and adjusted HRs with 95% CIs for mortality related to clinical parameters and fatty acid oxidation-related expression of enzymes. T3/T4 case (*n* = 58).

Factor	Crude HR (95% CI)	*p*-Value	Adjusted HR (95% CI)	*p* -Value
Age, per 10 years	1.46 (1.01–2.12)	0.047 *	1.92 (1.16–3.18)	0.012 *
Pretreatment fasting sugar: ≥100 vs. <100 mg/dL	0.67 (0.28–1.61)	0.369	0.41 (0.16–1.07)	0.069
FAS: 2~3+ vs. 0~1+	0.16 (0.02–1.19)	0.073	0.13 (0.02–1.00)	0.050
MCAD: 3+ vs. 0~2+	1.00 (0.42–2.42)	0.997	1.16 (0.46–2.92)	0.757
LCAD: 2~3+ vs. 0~1+	0.24 (0.03–1.78)	0.162	0.09 (0.01–0.85)	0.035 *
VLCAD: 2~3+ vs. 0~1+	1.51 (0.55–4.16)	0.425	0.99 (0.32–3.11)	0.989
HADHA: 3+ vs. 0~2+	1.22 (0.41–3.66)	0.723	0.44 (0.13–1.50)	0.187

VIF values: Age: 1.70, Pretreatment fasting sugar: 1.15, FAS: 1.28, MCAD: 1.65, LCAD: 1.38, VLCAD: 1.06, HADHA: 1.40. HR based on age as a continuous variable. * *p* < 0.05; *n*: number; HR: hazard ratio; CI: confident interval; T: tumor; N: node; FAS: fatty acid synthase; MCAD: medium-chain acyl-CoA dehydrogenase; LCAD: long-chain acyl-CoA dehydrogenase; VLCAD: very-long-chain acyl-CoA dehydrogenase; HADHA: hydroxyacyl-CoA dehydrogenase/3-ketoacyl-CoA thiolase/enoyl-CoA hydratase; VIF: variance inflation factor.

## Supplementary Materials

The following are available online at https://www.mdpi.com/1422-0067/21/18/6851/s1.

## Abbreviations

ACAD	acyl-CoA dehydrogenase
ACC	acetyl-CoA carboxylase
CI	confidence interval
CPT1	carnitine palmitoyltransferase 1
FAO	fatty acid oxidation
FAS	fatty acid synthase
HADHA	hydroxyacyl-CoA dehydrogenase/3-ketoacyl-CoA thiolase/enoyl-CoA hydratase
HCC	hepatocellular carcinoma
HPV	Human Papillomavirus
HR	hazard ratio
IHC	immunohistochemistry
IQR	interquartile range
LCAD	long-chain acyl-CoA dehydrogenase
MCAD	medium-chain acyl-CoA dehydrogenase
OS	overall survival
pACC1	phosphorylated acetyl-CoA carboxylase 1
SCCHN	squamous cell carcinoma of the head and neck
TNM	tumor-node-metastasis
VLCAD	very-long-chain acyl-CoA dehydrogenase
VIF	variance inflation factor
