# Supplementary material for: Prognostic Value of the Overexpression of Fatty Acid Metabolism-Related Enzymes in Squamous Cell Carcinoma of the Head and Neck
Source: Int J Mol Sci. 2020 Sep 18;21(18):6851. doi: 10.3390/ijms21186851 (PMC7559281; doi:10.3390/ijms21186851)
Supplement: Supplementary file 1 [file ijms-21-06851-s001.pdf]

Table S1. Correlations between the patients' age and clinical characteristics (n = 102).

Factor	Age <60 (n = 74)	Age ≥60 (n = 28)	p-value [a]
**Stage I/II** n (%)	17 (22.97%)	6 (21.43%)	1.00
**Pretreatment fasting sugar** median (IQR)	96.00 (90.00, 106.00)	100.50 (86.00, 112.00)	1.00
**Pretreatment cholesterol** median (IQR)	178.00 (144.00, 204.00)	194.00 (159.00, 221.00)	0.54
**Pretreatment triglyceride** median (IQR)	105.00 (84.00, 136.00)	109.00 (83.50, 130.00)	0.87
**FAS** (2~ 3+) n (%)	10 (13.51%)	4 (14.29%)	1.00
**CPT1**(3+) n (%)	14 (18.92%)	0 (0.00%)	0.01*
**MCAD** (3+) n (%)	24 (32.43%)	11 (39.29%)	0.68
**LCAD** (2~3+) n (%)	13 (17.57%)	3 (10.71%)	0.55
**VLCAD** (2~3+) n (%)	11 (14.86%)	3 (10.71%)	0.75
**HADHA** (3+) n (%)	6 (8.11%)	6 (21.43%)	0.09

[a] χ^2 test or Fisher's exact test for categorical variables / Mann-Whitney U test for continuous variables. * $p < 0.05$; n: number; IQR: interquartile range; T: tumor; N: node; FAS: fatty acid synthase; CPT1: carnitine palmitoyl transferase 1; MCAD: medium-chain acyl-CoA dehydrogenase; LCAD: long-chain acyl-CoA dehydrogenase; VLCAD: very-long-chain acyl-CoA dehydrogenase; HADHA: hydroxyacyl-CoA dehydrogenase/3-ketoacyl-CoA thiolase/enoyl-CoA hydratase.

Table S2. Correlations between N stage and patients' characteristics (n = 102).

Factor	Node-negative (n = 49)	Node-positive (n = 53)	p-value [a]
**Age** median (IQR)	52.70 (45.13, 60.91)	50.19 (45.63, 61.03)	0.47
**Pretreatment fasting sugar** median (IQR)	95.00 (91.00, 104.00)	100.00 (88.00, 110.00)	0.32
**Pretreatment cholesterol** median (IQR)	175.00 (15.00, 202.00)	187.00 (154.00, 208.00)	0.50
**Pretreatment triglyceride** median (IQR)	107.00 (83.00, 128.00)	106.00 (84.00, 145.00)	0.81
**FAS** (2~3+) n (%)	8 (16.33%)	6 (11.32%)	0.66
**CPT1**(3+) n (%)	7 (14.29%)	7 (13.21%)	1.00
**MCAD** (3+) n (%)	14 (28.57%)	21 (39.62%)	0.33
**LCAD** (2~3+) n (%)	5 (10.20%)	11 (20.75%)	0.23
**VLCAD** (2~3+) n (%)	7 (14.29%)	7 (13.21%)	1.00
**HADHA** (3+) n (%)	7 (14.29%)	5 (9.43%)	0.65

[a] χ^2 test or Fisher's exact test for categorical variables / Mann-Whitney U test for continuous variables. * $p < 0.05$; n: number; IQR: interquartile range; T: tumor; N: node; FAS: fatty acid synthase; CPT1: carnitine palmitoyl transferase 1; MCAD: medium-chain acyl-CoA dehydrogenase; LCAD: long-chain acyl-CoA dehydrogenase; VLCAD: very-long-chain acyl-

CoA dehydrogenase; HADHA: hydroxyacyl-CoA dehydrogenase/3-ketoacyl-CoA thiolase/enoyl-CoA hydratase.

Table S3. Correlations between the clinical HPV P16 and patients' characteristics (*n* = 102).

Factor	P16 (-) (*n* = 89)	P16 (+) (*n* = 13)	*p*-value [a]
**Age** median (IQR)	52.64 (45.36, 61.33)	51.24 (43.52, 52.28)	0.316
**Pretreatment fasting sugar** median (IQR)	96.00 (87.00, 108.00)	101.00 (95.00, 105.00)	0.408
**Pretreatment cholesterol** median (IQR)	184.00 (152.00, 206.00)	175.00 (154.00, 195.00)	0.564
**Pretreatment triglyceride** median (IQR)	109.00 (84.00, 136.00)	95.00 (86.00, 114.00)	0.388
**FAS** (2~ 3+) *n* (%)	11 (12.36%)	3 (23.08%)	0.381
**CPT1**(3+) *n* (%)	14 (15.73%)	0 (0.00%)	0.206
**MCAD** (3+) *n* (%)	32 (35.96%)	3 (23.08%)	0.534
**LCAD** (2~3+) *n* (%)	14 (15.73%)	2 (15.38%)	1.000
**VLCAD** (2~3+) *n* (%)	10 (11.24%)	4 (30.77%)	0.077
**HADHA** (3+) *n* (%)	12 (13.48%)	0 (0.00%)	0.355

[a] χ^2 test or Fisher's exact test for categorical variables / Mann-Whitney U test for continuous variables. * $p < 0.05$; *n*: number; IQR: interquartile range; T: tumor; N: node; FAS: fatty acid synthase; CPT1: carnitine palmitoyl transferase 1; MCAD: medium-chain acyl-CoA dehydrogenase; LCAD: long-chain acyl-CoA dehydrogenase; VLCAD: very-long-chain acyl-CoA dehydrogenase; HADHA: hydroxyacyl-CoA dehydrogenase/3-ketoacyl-CoA thiolase/enoyl-CoA hydratase; HPV: Human papillomavirus

Table S4. Correlations between Ki-67 expression and patients' characteristics (*n* = 102).

Factor	Ki-67 < 10% (*n* = 94)	Ki-67 ≥ 10% (*n* = 8)	*p*-value [a]
**Age** median (IQR)	51.96 (45.13, 61.03)	55.51 (47.72, 61.07)	0.467
**Pretreatment fasting sugar** median (IQR)	96.00 (88.00, 106.00)	100.50 (88.00, 126.00)	0.588
**Pretreatment cholesterol** median (IQR)	182.00 (150.00, 206.00)	179.00 (156.00, 190.50)	0.746
**Pretreatment triglyceride** median (IQR)	106.50 (84.00, 132.00)	102.00 (80.00, 208.50)	0.990
**FAS** (2~3+) *n* (%)	13 (13.83)	1 (12.50)	1.000
**CPT1**(3+) *n* (%)	13 (13.83)	1 (12.50)	1.000
**MCAD** (3+) *n* (%)	33 (35.11)	2 (25.00)	0.712
**LCAD** (2~3+) *n* (%)	13 (13.83)	3 (37.50)	0.109
**VLCAD** (2~3+) *n* (%)	11 (11.70)	3 (37.50)	0.077
**HADHA** (3+) *n* (%)	12 (12.77)	0 (0.00)	0.591

[a] χ^2 test or Fisher's exact test for categorical variables / Mann-Whitney U test for continuous variables. * $p < 0.05$; *n*: number; IQR: interquartile range; T: tumor; N: node; FAS: fatty acid synthase; CPT1: carnitine palmitoyl transferase 1; MCAD: medium-chain acyl-CoA dehydrogenase; LCAD: long-chain acyl-CoA dehydrogenase; VLCAD: very-long-chain acyl-

CoA dehydrogenase; HADHA: hydroxyacyl-CoA dehydrogenase/3-ketoacyl-CoA thiolase/enoyl-CoA hydratase.
